# Visual Intratumor Heterogeneity and Breast Tumor Progression

**DOI:** 10.3390/cancers16132294

**Published:** 2024-06-21

**Authors:** Yao Li, Sarah C. Van Alsten, Dong Neuck Lee, Taebin Kim, Benjamin C. Calhoun, Charles M. Perou, Sara E. Wobker, J. S. Marron, Katherine A. Hoadley, Melissa A. Troester

**Affiliations:** 1 Department of Statistics and Operations Research, University of North Carolina at Chapel Hill, Chapel Hill, NC 27599, USA; yaoli@email.unc.edu (Y.L.); taebinkim@unc.edu (T.K.); marron@unc.edu (J.S.M.); 2 Department of Epidemiology, University of North Carolina at Chapel Hill, Chapel Hill, NC 27599, USA; sarahvan@email.unc.edu; 3 Department of Biostatistics, University of North Carolina at Chapel Hill, Chapel Hill, NC 27599, USA; east90@live.unc.edu; 4 Department of Pathology and Laboratory Medicine, University of North Carolina at Chapel Hill, Chapel Hill, NC 27599, USA; ben.calhoun@unchealth.unc.edu (B.C.C.); chuck_perou@med.unc.edu (C.M.P.); sara_wobker@med.unc.edu (S.E.W.); 5 UNC Lineberger Comprehensive Cancer Center, University of North Carolina at Chapel Hill, Chapel Hill, NC 27599, USA; hoadley@med.unc.edu; 6 Department of Genetics, University of North Carolina at Chapel Hill, Chapel Hill, NC 27599, USA

**Keywords:** intratumor heterogeneity, breast cancer, machine learning

## Abstract

**Simple Summary:**

This study investigates the role of visual intratumor heterogeneity (ITH) in breast cancer progression. By analyzing histologic images from the Carolina Breast Cancer Study (CBCS) and the Cancer Genome Atlas Breast Invasive Carcinoma (TCGA-BRCA) data using advanced image processing and machine learning techniques, we developed a measure of tumor heterogeneity based on visual features. Our findings indicate that tumors with low visual heterogeneity exhibited a higher risk of recurrence and were more likely to come from patients whose tumors comprised of only one subclone or had a TP53 mutation. Conversely, high visual heterogeneity was correlated with a more favorable prognosis. These results suggest that visual heterogeneity provides complementary information to molecular markers. A comprehensive understanding of both the visual and molecular aspects of heterogeneity has the potential to offer novel insights for treatment strategies.

**Abstract:**

High intratumoral heterogeneity is thought to be a poor prognostic indicator. However, the source of heterogeneity may also be important, as genomic heterogeneity is not always reflected in histologic or ‘visual’ heterogeneity. We aimed to develop a predictor of histologic heterogeneity and evaluate its association with outcomes and molecular heterogeneity. We used VGG16 to train an image classifier to identify unique, patient-specific visual features in 1655 breast tumors (5907 core images) from the Carolina Breast Cancer Study (CBCS). Extracted features for images, as well as the epithelial and stromal image components, were hierarchically clustered, and visual heterogeneity was defined as a greater distance between images from the same patient. We assessed the association between visual heterogeneity, clinical features, and DNA-based molecular heterogeneity using generalized linear models, and we used Cox models to estimate the association between visual heterogeneity and tumor recurrence. Basal-like and ER-negative tumors were more likely to have low visual heterogeneity, as were the tumors from younger and Black women. Less heterogeneous tumors had a higher risk of recurrence (hazard ratio = 1.62, 95% confidence interval = 1.22–2.16), and were more likely to come from patients whose tumors were comprised of only one subclone or had a TP53 mutation. Associations were similar regardless of whether the image was based on stroma, epithelium, or both. Histologic heterogeneity adds complementary information to commonly used molecular indicators, with low heterogeneity predicting worse outcomes. Future work integrating multiple sources of heterogeneity may provide a more comprehensive understanding of tumor progression.

## 1. Introduction

Breast tumor evolution has been described using an evolutionary model, wherein successive selective sweeps result in more aggressive phenotypes [1,2]. Some have hypothesized that heterogeneity across a tumor mass would offer a selective advantage, with some clones having ‘fitness’ to resist cell death or to increase proliferation. Accordingly, at least one previous study has suggested that intratumor heterogeneity (as measured by genetic markers or ER status) is associated with poor prognosis [3,4]. However, the scope and timing of observed heterogeneity may also have significance for outcomes. Early in cancer development, heterogeneity provides a wide range of phenotypes that permit evolutionary selection [5]; later, heterogeneity potentially preserves therapy-sensitive cells and is the basis for ‘adaptive therapy’ regimens [6].

In addition to temporal dynamics, heterogeneity can occur on multiple scales (e.g., genomic, epigenetic, microenvironmental). For instance, different tumor cell populations may carry distinct mutations and copy number alterations [7], while the same mutation can give rise to disparate phenotypes depending on the cell of origin [8]. Assessing heterogeneity at multiple levels is therefore important for understanding potential effects on cancer behavior and outcome. Previous research has primarily focused on measurement of heterogeneity at the cellular and genomic levels, in large part because spatial resolution cannot be resolved from bulk sequencing [9,10]. Single-cell and spatially resolved transcriptomic studies provide an alternative, and have been used to map phenomena such as immune cell infiltration [11,12], evolution of copy number instability [13], and cell–cell interactions within tumors [14], but they remain technically challenging and difficult to apply in large samples. Thus, there remains a need for additional tools that can measure broader, tissue-level scales of heterogeneity from routinely collected tumor data, such as hematoxylin and eosin (H&E)-stained tissue microarray (TMA) core images. In this analysis, we aimed to (1) develop a flexible, visual (as opposed to molecular) indicator of tumor heterogeneity and (2) explore associations between histologic heterogeneity, clinical characteristics, and outcomes. Additionally, most studies of heterogeneity have focused on genetic or molecular markers of heterogeneity, and few studies have evaluated the histologic/visual heterogeneity of tumors as a function of stage and other prognostic indicators. Histologic heterogeneity reflects a different scale of tumor evolution relative to molecular assessments, providing an indication of the spatial distribution of evolving phenotypes, and it may therefore add value to other assessments of heterogeneity.

To evaluate histologic heterogeneity, we studied breast cancer hematoxylin and eosin (H&E)-stained tissue microarray (TMA) core images using a population-based sample of incident invasive breast cancers. Using data from the Carolina Breast Cancer Study (CBCS), a population-based study representing the range of tumor stage and molecular subtype in North Carolina and oversampling young women and Black women to ensure representation of these two understudied groups, we evaluated multiple TMA images from each patient (n=3 cores per patient on average, n=1655 patients) to identify histologic heterogeneity between cores. Using computer vision tools, we quantitatively characterized visual intraiumor heterogeneity (visual ITH) using a Normalized Merge Level (NML) score that reflects dissimilarity across image features. High NML scores can be interpreted as a low level of visual similarity within a single patient relative to the level of similarity between different patients. A binary variable, visual ITH group (homogeneous vs. heterogeneous), was created based on the NML score to evaluate how visual ITH related to clinical variables, such as recurrence, ER status, and tumor grade, and molecular indicators of heterogeneity, such as number of subclones.

## 2. Materials and Methods

### 2.1. Study Population

The study population of the Carolina Breast Cancer Study Phase 3 (CBCS3) was sampled using Rapid Case Ascertainment with oversampling for Black women and women under the of age 50, both of whom tend to be under-represented in breast cancer research data. All cases (aged 20–74) were diagnosed with first, primary invasive breast cancer between 2008 and 2013. The study population and methods have been described in detail elsewhere [15,16,17,18,19,20]. The current analytic sample was restricted to cases with at least two pre-treatment H&E-stained 1 mm cores on tissue microarrays (TMA), with most cases having three TMA cores (n=3 cores per case, on average). A total of 1655 unique patients with 5907 TMA cores in CBCS3 were included. All study procedures were approved by the University of North Carolina (UNC) School of Medicine Institutional Review Board, and patients provided written informed consent.

Recurrence data were available for CBCS3. Recurrence-free survival (RFS) was defined as the time between date of diagnosis to first local, regional, or distant recurrent breast cancer and verified through medical record review. Follow-up was complete through October 2019, with at least a 5-year follow-up for all women. Among the 1655 participants, 171 recurrences were identified, with missing recurrence observations for 363 participants.

We also applied visual ITH analysis to the Cancer Genome Atlas Breast Invasive Carcinoma (TCGA-BRCA) data, which have been described in previous studies [21,22]. A total of 1102 patients, which have both whole-slide images and clinical data available, were included in the analysis. For each patient, a breast pathologist looked at the corresponding whole-slide image and identified 2 to 8 ‘virtual cores’, each of which is approximately the same size as the CBCS TMA core. Therefore, the analysis involved 1102 patients with 6556 virtual TMA cores. All TCGA samples were processed under the approval of the respective Institutional Review Boards, and patients provided written informed consent. The results shown here are in whole or part based upon data generated by the TCGA Research Network: https://www.cancer.gov/tcga (accessed on 4 February 2022).

### 2.2. Color Normalization and Image Segmentation

**Figure 1:**
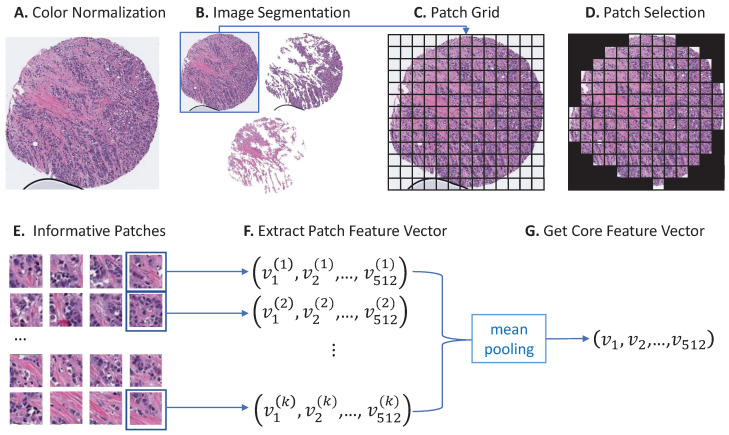
Pipeline of core feature extraction. (**A**) The first step in this pipeline is color normalization, which uses the method proposed in [23] to remove undesirable variations in the core images. (**B**) The normalized core images were then segmented [24] into three types of core images: original, epithelium, and stroma. (**C**) Since the core images are large, directly dealing with them is not feasible. Each core image was divided into smaller patches of 200×200 pixel size in this step. (**D**,**E**) Patches with more than 90% background and patches with artifacts were removed to keep only informative patches. (**F**) Convolutional Neural Networks (VGG16 [26]) were used to extract 512-dimensional feature vectors from the patches. (**G**) Feature vectors of all patches of a core were averaged to obtain one feature vector for each core image.

Color normalization [23] (see Figure 1A) was first applied to standardize the stain color intensity of the TMA core images because many factors (e.g., different patient populations, different scanners, different staining techniques) can cause artifacts that are undesirable in building a generalizable machine learning model. The appearance of the tissue samples can vary greatly without normalization, making it difficult for a model trained on one cohort to generalize to others.

An image segmentation method [24] was then applied using QuPath 0.3.2 [25] to classify each pixel of a TMA core into one of the three classes: epithelium, stroma, or background. Image segmentation enables the study of visual characteristics of different tissue types (especially epithelium and stroma). Adipocyte tissue was combined with background as one class when applying segmentation. The segmentation resulted in three sets of images for each core: (1) the original image with background removed; (2) an epithelial region of interest image; and (3) a stroma-only region of interest with epithelium excluded. Examples of the three types of images we used in this study are shown in Figure 1B. To avoid confusion, when we refer to the tumor, we mean the epithelium-enriched but stroma- and immune-inclusive components of cellularity. When we refer to the epithelium, we are specifically indicating those components of the tumor that are epithelial in morphology.

### 2.3. Core Feature Extraction

Convolutional Neural Networks (CNNs) are widely applied to extract image features from medical images and have been successful in many downstream tasks compared to cell-by-cell morphology features [27,28,29,30,31,32]. However, directly applying CNN models to core images is infeasible due to the large size (2600×2600 pixels on average per core). Thus, we split the cores into smaller 200×200 patch pixels (Figure 1C). Patches with more than 90% background and patches with artifacts (e.g., bubbles, tissue folding, etc., such as Figure 1C) were removed (Figure 1D,E). Remaining patches were input into a CNN model, VGG16 [26], to extract feature vectors. The VGG16 model was pre-trained on a large image dataset: ImageNet [33]. Each patch was summarized as one 512-dimensional feature vector by the VGG16 model, and the feature vector of each core was generated by averaging the feature vectors of all the patches of the core (Figure 1F,G). Therefore, each core image was represented by a 512-dimensional feature vector after the feature extraction process. The same procedure was applied to all three types of core images (original, epithelium, and stroma) to extract core feature vectors.

### 2.4. Feature Clustering

To view the visual ITH, the degree to which the tumor was visually dissimilar across different spatially sampled regions, we applied a hierarchical clustering method [34] on the extracted core features and set the total number of clusters to the number of patients in the study population (n=1655). Each core was assigned a cluster label by the clustering method based on the similarity of core features. We used Euclidean distance with Ward’s linkage for the analysis [35].

Patients were defined as having low visual ITH if the core features were similar to those of the same patient (low within-person variability), but different from those of a different patient (high between-person variability). If the histologic features of a patient are visual intratumor heterogeneous, the clustering result will not aggregate cores from the same individual patient, showing random clustering patterns instead. These patients had high visual ITH.

### 2.5. Measure of Visual Intratumor Heterogeneity

**Figure 2:**
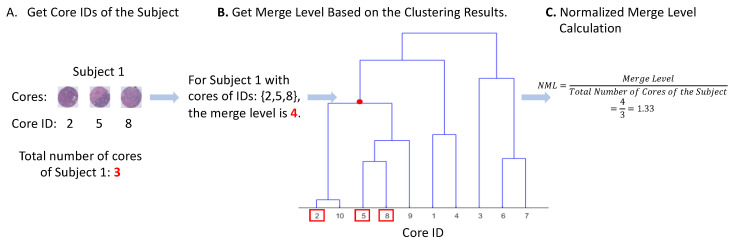
A toy example of Normalized Merge Level (NML) calculation of a patient. Hierarchical clustering method [34] was first applied to the core features to divide the cores into 1655 clusters, which is the same as the total number of patients in CBCS3. Clustering results were summarized in a clustering tree. To obtain the NML of a patient, (**A**) core IDs or the patient were first identified. (**B**) Based on the core IDs, the merge level (level of the tree where all cores of the patient merge) of the patient can be found via the clustering tree. (**C**) NML was calculated by dividing the merge level by the total number of cores of the patient.

To quantify visual ITH across cores for a patient, we developed a Normalized Merge Level (NML) score. This method is based on hierarchical clustering results and can be viewed as a tree, with each leaf node representing one core and the root node representing one big cluster that contains all the cores. The clustering result is a specific level of the tree that has 1655 (the number of patients in the CBCS3 data) clusters. If a patient has homogeneous histologic features, its cores will merge at a low level of the tree, indicating that the core features of the patient are highly similar. However, if the tumor of a patient is visually heterogeneous, the cores will merge at a relatively high level of the tree. Therefore, the merge level of a patient, which is defined as the level of the tree where all cores of a patient merge, can be a measure of visual ITH, with a high merge level value representing heterogeneity and a low value indicating homogeneity (see a toy example in Figure 2).

For a merge level with different numbers of cores per patient, it is important to address how criterion merge levels vary with different numbers of cores. Patients with more cores tend to have larger merge levels, while patients with fewer cores tend to have smaller ones, which causes bias in the criterion in measuring visual ITH. To alleviate the bias, we normalized the merge level criterion by dividing by the number of cores of the patient, which we refer to here as the Normalized Merge Level (NML). Based on the clustering tree, the NML score of each patient can be calculated to represent its visual ITH score, with higher visual ITH representing more heterogeneity.

Since each core includes a different representation of different tissue types (Figure 1B), the clustering method was applied separately to original images as well as QuPath segmented epithelial and stroma components to calculate three visual ITH NML scores for each patient. These visual ITH scores were the basis for our visual ITH group variable, a binary variable based on visual ITH score.

### 2.6. Measure of Molecular Heterogeneity

DNA from 344 formalin-fixed paraffin-embedded tumor cores and paired normal blood samples (where available) was isolated and sequenced using a custom Agilent panel, targeting exons of 1200 genes (UNCSeq) at an average of 400X depth. Paired-end FASTQ files were generated from runs on Illumina sequencers (NovaSeq, HiSeq, NextSeq, or MiSeq-Nano) using standard protocols at UNC, and then aligned to the human reference genome 38 (GrCh38) with BWAmem. After sorting and indexing, we realigned paired tumor and normal BAMs with ABRA2 [36] and calculated somatic variants with Mutect2 [37], Strelka2 [38], and Cadabra [36]. We removed any variants covered by fewer than 15 total reads, supported by <5 alternate reads, or with a variant allele frequency <15%, as the probability of false positive calls is higher for such variants. We also calculated allele-specific copy number with cnvkit [39]. Aligned BAM files from normal samples were used to generate a pooled reference of expected sequencing coverage across target regions; then, we compared this with tumor coverage to estimate copy number. Change-points were detected using circular binary segmentation. Tumors with a high standard deviation (>2) and median absolute deviation (>1) read coverage were removed from the copy number analyses (N = 33), leaving 344 tumors profiled for mutations and 311 for copy number.

We hypothesized that tumors with low visual ITH would also show low molecular ITH, defined as being comprised of fewer tumor subclones. Therefore, we estimated tumor subclone number and composition using PyClone-VI [40], a Bayesian algorithm which clusters mutations into distinct subclonal populations according to variant allele frequency and allele-specific copy number. After mapping each mutation to the overlapping copy number segment from the same sample, we clustered mutations with PyClone-VI and extracted the total number of clusters observed in each sample. Because few samples had more than two subclones, we dichotomized clonality as ‘one subclone’ or ‘more than one subclone’. In addition, given our interest in the TP53 gene as a key driver of tumor heterogeneity and clonal expansion, we categorized tumors as ‘TP53 mutation’ versus ‘no TP53 mutation’.

### 2.7. Statistical Analysis

The patients in this study population were divided into two groups based on the visual ITH score: low visual ITH versus high visual ITH (heterogeneous). Kernel density estimation was applied to estimate the distribution of the visual ITH scores, and then a cutoff was selected to divide the scores into two groups. If there was an obvious bimodal pattern, the cutoff was selected as the value that separates the two modes. If not, the median was selected as the cutoff. For CBCS3, an obvious bimodal pattern was observed, and the cutoff was set at 3.75. For TCGA-BRCA, no such pattern was observed, so the median cutoff of 1.28 was used. The relationship between the visual ITH group of a patient and their clinical variables were analyzed by using a generalized linear model. Three different visual ITH binarized variables were created based on the three types of core images (original, epithelium, and stroma) and assessed with the following statistical analyses.

Generalized linear models were used to calculate Relative Frequency Differences (RFDs) and corresponding 95% confidence intervals (CIs) as measures of association between visual ITH groups and variables of interest. RFDs were estimated based on a general linear model with binomial distribution and identity link, and were interpretable as the percentage difference in heterogeneity between index and referent groups. The following variables were studied in association with visual ITH group: age at diagnosis (≥50, <50), race (self-reported Black, non-Black (>98% white)), tumor grade (low, intermediate, high), PAM50 risk of recurrence (ROR) (low, medium, high), tumor size (≤2 cm, >2 cm), PAM50 intrinsic breast cancer subtype (Luminal A, Luminal B, HER2-enriched, basal-like, normal-like) [41], ER status (negative, positive), node status (negative, positive), and previously identified immune subtypes [42]. Multivariate models were adjusted for age and race, or the models were adjusted for node status, tumor size, ER status, and tumor grade. Kaplan—Meier curves were used to compare mean time to recurrence between the visual ITH groups. Hazard ratios (HRs) and 95% CIs were calculated using Cox proportional hazard models. The assumption of proportionality was assessed via the Wald *p*-value. Associations between visual ITH and molecular indicators (subclone number and TP53 mutation) were also assessed but, given the smaller number of patients with available DNA data, we used logistic regression to ensure model convergence.

To evaluate the generalizability of this approach to a different dataset, we calculated the visual ITH scores for patients using TCGA-BRCA data. The scores were calculated based on histopathology for 1102 patients, with 6556 representative core TMA images. Visual ITH scores based on original TMA images were generated for statistical analysis to compare with the corresponding results based on CBCS3 data. Molecular heterogeneity was again defined based on the number of tumor subclones (one subclone vs. more than one; with all mutations from whole-exome sequencing clustered in PyClone-VI) and TP53 mutation status (mutation vs. no mutation). All statistical analyses were performed in R version 4.1.1.

## 3. Results

### 3.1. Visual ITH, Patient, and Tumor Characteristic

**Figure 3:**
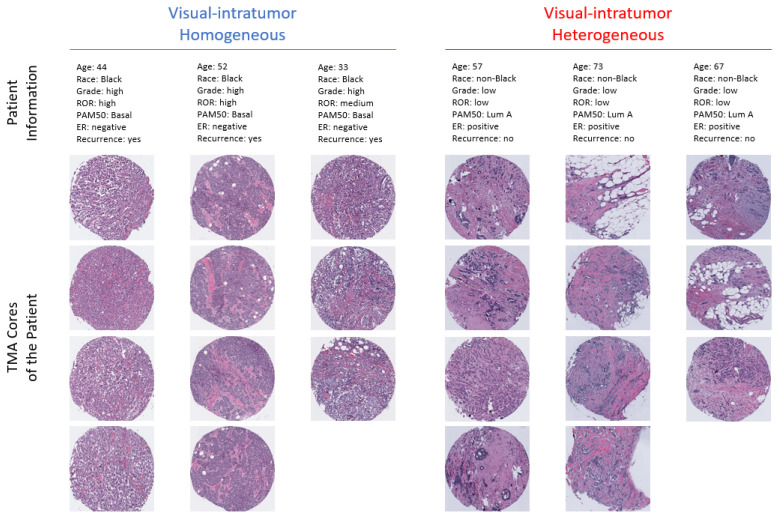
Examples of visual intratumor homogeneous and heterogeneous patients and their corresponding TMA cores. The left panel shows the information and TMA cores of three patients from the visual intratumor homogeneous group. The right panel shows the information and TMA cores of three patients from the visual intratumor heterogenous group.

We calculated visual ITH scores based on histopathology for 1655 patients, with 5907 representative TMA images from CBCS3. We extracted image features from these core images three times, once for the original image and separately for masked images that contained only epithelial or stromal compartments. The visual ITH score used tree-cluster distance, adjusted for number of cores per patient. Examples of participant cores with high visual ITH scores (heterogeneous group) and low visual ITH scores (homogeneous group) are shown in Figure 3. There is visual similarity between cores of the same patient in the left panel, while there is no obvious similarity on the right. We found that in original images, 729 (44%) had visually heterogeneous tumors, with similar results for epithelium (45%) and a slightly higher frequency of visual ITH for stroma (51%).

**Figure 4:**
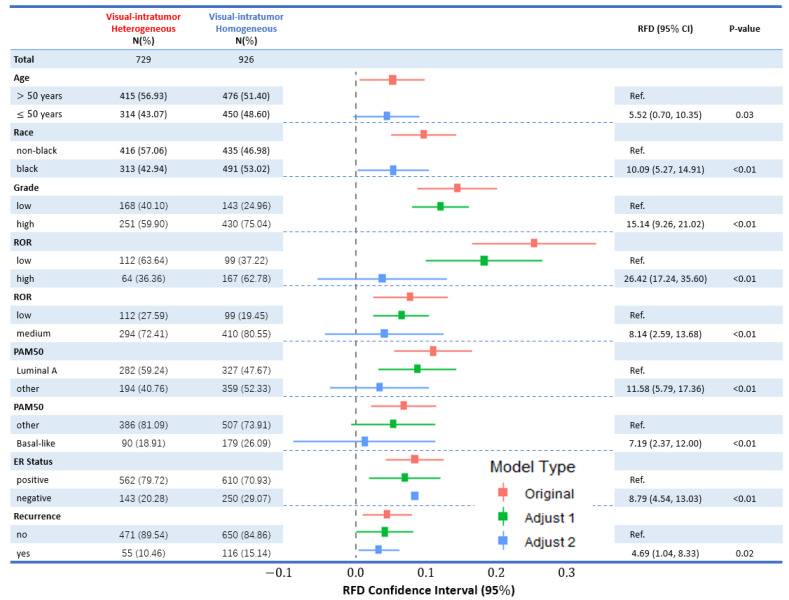
Associations between patient and tumor characteristics and visual ITH group (H&E) in CBCS3. Forest plot displays Relative Frequency Differences (RFDs) and 95% CIs for patient age, race, tumor grade, ROR, PAM50 type, ER status, and recurrence across visual intratumor homogeneous and heterogeneous groups. Unadjusted (original) RFDs are shown in red in the middle plot and the corresponding statistics are available in the last two columns. The RFDs and 95% CIs for models adjusted for age and race (Adjust 1) are shown in green, while the models adjusted for tumor grade, ER status, node status, and tumor size (Adjust 2) are shown in blue in the middle plot. Referent groups for each individual model are indicated in the figure, and sample size (N) and percentages are listed. Referent group: visual intratumor heterogeneous group for all models. Note that adjusted results (green bars) do not show results for age and race, nor do blue bars for tumor grade, ER status, node status and tumor size, as these variables were part of the adjustment set and not main effects.

Visual ITH from original images was evaluated in association with patient age at diagnosis, race, tumor grade, ROR, PAM50, ER, and recurrence in CBCS3 (Figure 4). The frequencies, percentages, Relative Frequency Differences (RFDs), and corresponding 95% CIs were calculated based on models without adjustment (reduced), and after adjusting for age and race (Adjust 1); and tumor grade, ER status, node status, and tumor size (Adjust 2). Low visual ITH score was associated with younger age, Black race, high tumor grade, high ROR, basal-like PAM50 type, negative ER status, and recurrence. In the reduced model, these associations were significant, though some were attenuated after adjusting for age and race and after adjusting for tumor grade, ER status, node status and tumor size. The associations were mostly significant when adjusted for race and age (Adjust 1) models, while the patterns were mostly not significant when further adjusted for grade, ER, node and size models (Adjust 2), suggesting that visual ITH is a correlate of these factors and is not independent.

We performed sensitivity analyses to see if the results differed by the components of core images (stroma and epithelium). Visual ITH based on epithelium and stroma had a similar pattern of association with original image visual ITH (i.e., low visual ITH was associated with younger age, Black race, high tumor grade, high ROR, basal-like PAM50 type, negative ER status, and recurrence (Table 1)). Some associations were not significant when restricted to epithelium or stroma features, but the magnitude and direction of the association was unchanged. Adjusting for age and race did not substantially change the results (Table 2).

Applying the same analysis to TCGA-BRCA, we found that a higher proportion of participants 894 (71%) had high visual ITH. Visual ITH was evaluated in association with patient age at diagnosis, race, ROR, PAM50, ER, and recurrence (Table 3). The patterns of association were similar to CBCS3, with low visual ITH associated with younger age, Black race, high ROR, Basal-like PAM50 type, negative ER status and recurrence; however, the associations with binarized recurrence in TCGA were not significant. We conducted an analysis to study the association between immune subtype (low vs. high) for both the CBCS3 and TCGA-BRCA data. For CBCS3, the quiet and innate classes were combined to form the low group, while the adaptive class constituted the high group. We observed that visual intratumor homogeneity was associated with a higher adaptive immune response in CBCS3; however, this association was not observed in TCGA-BRCA. The results are presented in Table 3. The inconsistent results could be due to the fact that TCGA-BRCA has no obvious immune quiet samples, as the tumors were much more advanced. Therefore, the detected immune response in CBCS3 could result from a contrast between the quiet group and the adaptive group.

### 3.2. Visual ITH and Recurrence-Free Survival

**Figure 5:**
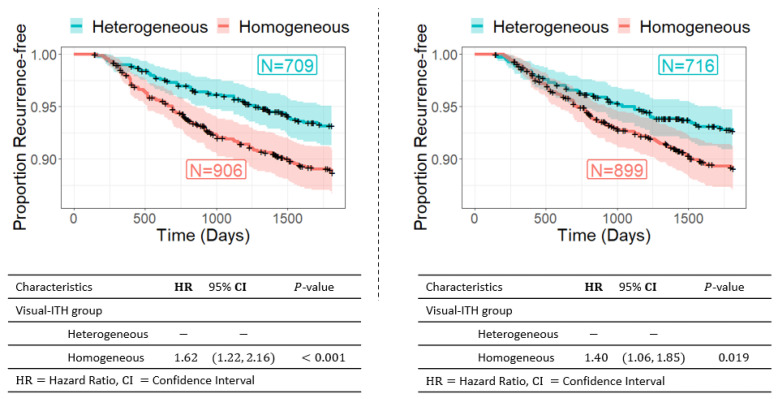
Visual intratumor homogeneous tumors have poorer prognosis than heterogeneous ones. Kaplan–Meier curves for 5-year recurrence free survival by original visual ITH (**A**) or epithelium visual ITH (**B**) from CBCS3 are shown along with hazard ratios (HRs) and 95% CIs estimated from Cox proportional hazards regression (Referent group: visual intratumor heterogeneous group).

**Figure A1:**
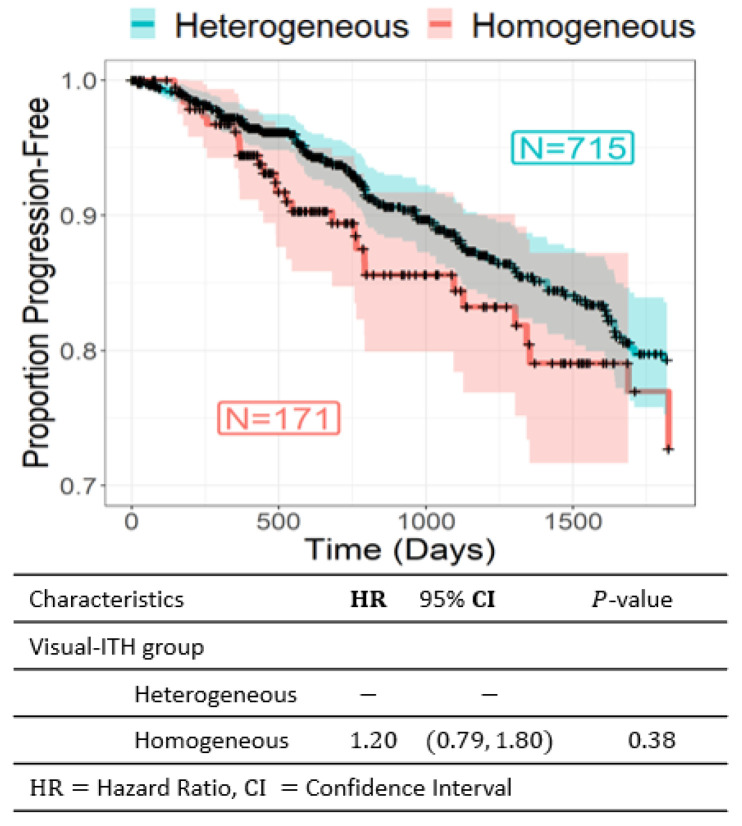
Kaplan–Meier curves for 5-year progression-free interval by visual ITH groups from TCGA-BRCA are shown, along with hazard ratios (HRs) and 95% CIs (Referent group: visual intratumor heterogeneous group) in the figure.

Comparing yes/no recurrence does not account for time to recurrence or loss to follow up, and we therefore also used time-to-event analyses to evaluate the relationship between visual ITH and recurrence. The CBCS3 identified 171 recurrences during the first 5 years of follow up in the study population. We assessed associations between visual ITH and recurrence using both Kaplan–Meier analyses and Cox proportional hazards models, without including treatment as a covariate. We found that lower visual ITH was associated with recurrence (HR = 1.62, 95% CI = 1.22–2.16). The association remained significant when restricting to the epithelial portions of images only, suggesting that higher visual ITH predicted better Recurrence-free survival (RFS) (Figure 5). A similar survival analysis for TCGA-BRCA using the progression-free interval was performed. We observed a tendency towards a similar pattern. Although the trend was in the same direction, with visually homogeneous tumors being more adverse, the association was not significant. The Kaplan–Meier curves are shown in Figure A1 in Appendix A.

### 3.3. Visual ITH, Genomic Instability, and Clonality by DNA Sequencing

To determine whether visual indicators of ITH reflected molecular differences, we assessed associations between visual ITH, number of tumor subclones (clonal vs. multi-clonal), and TP53 mutation status (Table 4). Low visual ITH was positively associated with tumor clonality in the original images (OR = 1.54, 95% CI = 0.98–2.45) and stromal segmented images (OR = 1.60, 95% CI = 1.01, 2.53). The association between epithelial-based visual ITH and clonality was null. Low visual ITH also showed a positive association with TP53 mutation status for original (OR = 1.98, 95% CI = 1.26–3.13) and stromal (OR = 1.72, 95% CI = 1.10, 2.70) components. Associations tended to be in the same direction, but were attenuated and non-significant when restricting to the epithelial compartment only, suggesting that the combination of epithelial and stromal features is an important contributor. A possible explanation for the association between good prognosis and stromal-based heterogeneity is that less aggressive tumors have higher proportions of stromal tissue [43], leading to greater potential to observe stromal histologic heterogeneity. In TCGA, where virtual cores were placed in epithelial regions, low-visual-ITH tumors were more likely clonal and TP53-mutated, though both associations were weak and null, as in estimates for CBCS epithelial regions.

## 4. Discussion

Tumor evolution over time is an important feature influencing cancer outcomes [2]. For example, ER+ breast cancers can develop endocrine resistance in response to therapeutic pressures, rendering endocrine therapy less effective [44], or accrue additional actionable mutations [45]. However, few studies have evaluated changes in tissue appearance over time (longitudinally or in cohorts as a function of stage or other markers of progression). Our results in a large, diverse cohort of cancer patients suggest that histologic heterogeneity/homogeneity can be measured in histopathologic images and that the development of homogeneity (i.e., low visual ITH) reflects a late stage of tumor evolution, possibly as a result of a selective sweep and a single cancer phenotype overtaking the histologic field. Tumors with low visual ITH, especially when defined by a combination of epithelium and stroma, had more advanced clinical characteristics, evidence of monoclonality, and were more likely to have TP53 mutations. Another key finding from our analysis was that associations between visual and molecular heterogeneity were strongest for visual components, including stroma. This suggests that the visual shape and boundary of a tumor may be indicators of molecular progression and differentiation, and emphasizes the importance of considering interactions between tumor and non-tumor cells.

Previous studies of intratumor heterogeneity have primarily emphasized molecular markers. For example, in studies by Keenan et al. and Pereira et al., ITH was defined according to Mutant-Allele Tumor Heterogeneity (MATH) score, which is based on the distribution of somatic variant allele frequencies [46,47]. These studies found higher genetic ITH to be a poor prognostic indicator, associated with higher risks of recurrence and more aggressive tumor features. While these results may seem to conflict with those from our study, differences may be explained by the scale at which heterogeneity was measured: namely, our study defined heterogeneity at a tissue-level scale rather than a molecular one. Molecular heterogeneity reflects the degree of genetic instability in the tumor; as evolution progresses, DNA repair defects accumulate, favoring error-prone repair and an accumulation of multiple low-frequency variant alleles [48]. This heterogeneity (i.e., late heterogeneity) may be secondary to an initial selective sweep for driver mutations like TP53. It has not yet been determined which molecular markers of heterogeneity, or whether early vs. late heterogeneity, have greater prognostic value, as opposed to those that reflect stochastic drift in the population of cancer cells. In contrast, visual or tissue-level heterogeneity reflects the spatial distribution and organization of both tumor and non-tumor compartments without direct consideration of the identity and phenotypes of component cells.

Prior work has suggested that as tumors progress, the level of spatial heterogeneity decreases while molecular heterogeneity increases [49], in principle, because tumor regions outcompete other cell types and outgrowth precludes the existence of more (visually) complex secondary structures. This conclusion is further supported by the fact that the observed associations in our study tended to be stronger when visual components included both epithelial and stromal regions, such that the score could consider interactions between multiple cell types. Therefore, histologic heterogeneity may represent an independent direction of heterogeneity, capturing tissue-level evidence of whether the tumor has taken on a more unified appearance. This also raises another recent hypothesis: as cancer cells evade homeostatic barriers, they take on more growth independence, possibly even acquiring changes that are ‘atavistic’ and reminiscent of unicellular organismal growth [50]. Indeed, many of the low-visual-ITH tumors are high-grade and show a very uniform distribution of cancer cells with high nuclear volume. Pending validation by further studies, our results suggest that among invasive tumors, those that have worse outcomes are more likely to be sampled after a single clone has emerged.

In our data, low-visual-ITH tumors were also more likely to harbor TP53 mutations and be comprised of only a single genetic clone, potentially reflecting the outcome of a selective sweep. These results were surprising, given that previous research has suggested that TP53 mutant tumors tend to have higher molecular intratumoral heterogeneity due to higher mutation burden [51] and chromosomal instability [52]; however, this may be partially explained by the fact that molecular measures are primarily based on epithelial tissue whereas visual measures also considered stromal components. Interactions with stroma are becoming increasingly emphasized in tumor prognostication, including in conjunction with TP53 mutation status [53,54]. This highlights the importance of cross-talk between tumor and non-tumor cells, some of which may be captured by higher-level visible structures. Similarly, our results may seem to conflict with previous analyses of heterogeneity in ER protein expression, which have suggested that high heterogeneity leads to worse outcomes [4]. However, heterogeneity in ER is difficult to disentangle from overall ER expression. That is, tumors with the highest ER positivity show low heterogeneity and are most likely to be responsive to estrogen-targeted therapy. Thus, we emphasize that the interpretation of heterogeneity likely depends on the methods of measurement. That is, it may vary according to the specific immunohistochemistry (IHC) of protein biomarkers and pathologic assessment by H&E. It is also important to consider that tumor histology is captured only at one moment in time, and the preceding changes (i.e., direct observation of temporal evolution) are unknown. Overall, this underscores the importance of considering the cellular and histologic context of a tumor, suggesting that heterogeneity at different temporal phases or modes of detection may have different implications.

The major strengths of this analysis include the use of a diverse population-based sample, integration of visual and molecular data, and the ability to address the influence of tumor composition by segmentation of epithelial and stromal tissue. A limitation of this analysis is that molecular heterogeneity measures were based on DNA from bulk sequencing of the tumors, which may underestimate clonal diversity relative to multi-region sequencing [55]. This may have particularly limited analyses in CBCS, where tumors carried fewer observed mutations due to the use of targeted sequencing. However, the similar proportions of multi-clonal and TP53 mutant tumors in the CBCS and TCGA data, and the agreement between molecular and visual heterogeneity (which, by definition, capture multiple tumor regions), suggests that molecular measures still capture relevant between-tumor variation.

## 5. Conclusions

In summary, our results highlight the value of considering histologic parameters in tandem with molecular markers of tumor evolution. With the advent of high-quality, high-depth sequencing, it is increasingly common to consider genetic heterogeneity; however, other ‘axes’ of evolution (such as histologic evolution or even organ-level evolution, as depicted by radiology images) may elucidate how tumors progress over time, leading to novel insights for treatment strategies.

## Figures and Tables

**Table 1:** Original visual intratumor heterogeneity measure shows the strongest associations with clinical characteristics. Comparison RFDs (95% CIs) based on three types of cores (original, epithelium, and stroma) are shown and were calculated using generalized linear models. Red: not significant. **Bold**: the biggest RFD among the three (original, epithelium, and stroma).

Variable	Ref.		Original (RFD, 95% CI)	Epithelium (RFD, 95% CI)	Stroma (RFD, 95% CI)
Age	>50	≤50	**5.52 (0.70, 10.35)**	1.88 (−2.95, 6.71)	1.73 (−3.07, 6.53)
Race	non-Black	Black	**10.09 (5.27, 14.91)**	6.97 (2.14, 11.80)	3.51 (−1.30, 8.33)
Grade	low	high	**15.14 (9.26, 21.02)**	8.82 (2.96, 14.69)	9.60 (3.86, 15.35)
ROR	low	high	**26.42 (17.24, 35.60)**	25.65 (16.55, 34.75)	16.74 (7.56, 25.92)
ROR	low	medium	8.14 (2.59, 13.68)	**10.91 (5.38, 16.45)**	6.23 (0.81, 11.66)
PAM50	Luminal A	other	11.58 (5.79, 17.36)	**12.80 (7.05, 18.56)**	8.29 (2.56, 14.01)
PAM50	other	basal-like	**7.19 (2.37, 12.00)**	7.06 (2.24, 11.87)	5.10 (0.26, 9.94)
ER Status	positive	negative	**8.79 (4.54, 13.03)**	7.04 (2.77, 11.30)	6.50 (2.20, 10.80)
Recurrence	no	yes	**4.69 (1.04, 8.33)**	4.48 (0.84, 8.13)	3.58 (−0.10, 7.27)

**Table 2:** Adjustment for age and race attenuate associations between visual intratumor heterogeneity and clinical characteristics. For each characteristic, unadjusted and race and age adjusted RFDs (95% CIs) based on epithelium and stroma cores are shown. Red: not significant.

Variable	Ref.		Epithelium (RFD, 95% CI)		Stroma (RFD, 95% CI)	
			Reduced	Adjusted	Reduced	Adjusted
Grade	low	high	8.82 (2.96, 14.69)	7.14 (3.19, 11.09)	9.60 (3.86, 15.35)	7.95 (4.10, 11.80)
ROR	low	high	25.65 (16.55, 34.75)	18.72 (10.49, 26.95)	16.74 (7.56, 25.92)	11.27 (4.04, 18.50)
ROR	low	medium	10.91 (5.38, 16.45)	9.65 (5.37, 13.93)	6.23 (0.81, 11.66)	5.75 (1.90, 9.60)
PAM50	Luminal A	other	12.80 (7.05, 18.56)	10.80 (5.06, 16.53)	8.29 (2.56, 14.01)	7.28 (1.78, 12.78)
PAM50	other	basal-like	7.06 (2.24, 11.87)	5.75 (−0.44, 11.94)	5.10 (0.26, 9.94)	4.37 (−1.80, 10.54)
ER Status	positive	negative	7.04 (2.77, 11.30)	5.93 (0.62, 11.25)	6.50 (2.20, 10.80)	5.88 (0.56, 11.19)
Recurrence	no	yes	4.48 (0.84, 8.13)	4.15 (0.10, 8.21)	3.58 (−0.10, 7.27)	3.39 (−0.91, 7.68)

**Table 3:** Comparisons of associations between visual intratumor heterogeneity and clinical characteristics in TCGA-BRCA and CBCS3. Relative Frequency Differences (RFDs) and 95% CIs for patient age, race, ROR, PAM50 type, ER status, immune infiltration class, and recurrence across visual intratumor homogeneous and heterogeneous groups were calculated using generalized linear models. For both datasets, the results were generated based on reduced models without adjustment. Referent groups for each individual model are indicated in the figure, and sample size (N) and percentages are listed. Referent group: visual intratumor heterogeneous group for all models.

	TCGA-BRCA				CBCS			
	**Visual-Intratumor**	**Visual-Intratumor**			**Visual-Intratumor**	**Visual-Intratumor**		
	**Heterogeneous**	**Homogeneous**	**RFD (95% CI)**	***p*** **-Value**	**Heterogeneous**	**Homogeneous**	**RFD (95% CI)**	***p*** **-Value**
	**N (%)**	**N (%)**			**N (%)**	**N (%)**		
**Total**	894	208			729	926		
**Age**								
>50 years	666 (74.58)	145 (70.05)	Ref.		415 (56.93)	476 (51.40)	Ref.	
≤50 years	227 (25.42)	62 (29.95)	4.53 (−2.33, 11.39)	0.18	314 (43.07)	450 (48.60)	5.52 (0.70, 10.35)	0.03
**Race**								
non-black	674 (86.19)	144 (78.69)	Ref.		416 (57.06)	435 (46.98)	Ref.	
black	108 (13.81)	39 (21.31)	7.50 (1.09, 13.91)	0.01	313 (42.94)	491 (53.02)	10.09 (5.27, 14.91)	<0.01
**ROR**								
low	208 (52.39)	29 (31.52)	Ref.		112 (63.64)	99 (37.22)	Ref.	
high	189 (47.61)	63 (68.48)	20.87 (10.18, 31.56)	<0.01	64 (36.36)	167 (62.78)	26.42 (17.24, 35.60)	<0.01
**ROR**								
low	208 (29.59)	29 (20.28)	Ref.		112 (27.59)	99 (19.45)	Ref.	
medium	495 (70.41)	114 (79.72)	9.31 (1.90, 16.71)	0.02	294 (72.41)	410 (80.55)	8.14 (2.59, 13.68)	<0.01
**PAM50**								
Luminal A	470 (52.63)	93 (44.93)	Ref.		282 (59.24)	327 (47.67)	Ref.	
other	423 (47.37)	114 (55.07)	7.70 (0.18, 15.23)	0.05	194 (40.76)	359 (52.33)	11.58 (5.79, 17.36)	<0.01
**PAM50**								
other	757 (84.77)	159 (76.81)	Ref.		386 (81.09)	507 (73.91)	Ref.	
Basal-like	136 (15.23)	48 (23.19)	7.96 (1.75, 14.17)	0.01	90 (18.91)	179 (26.09)	7.19 (2.37, 12.00)	<0.01
**ER Status**								
positive	677 (79.83)	144 (71.64)	Ref.		562 (79.72)	610 (70.93)	Ref.	
negative	171 (20.17)	57 (28.36)	8.19 (1.40, 14.98)	0.01	143 (20.28)	250 (29.07)	8.79 (4.54, 13.03)	<0.01
**Immune Class**								
low	488 (54.65)	117 (56.52)	Ref.		307 (75.25)	357 (60.92)	Ref.	
high	405 (45.35)	90 (43.48)	−1.87 (−5.63, 9.38)	0.63	101 (24.75)	229 (39.08)	14.32 (8.57, 20.08)	<0.01
**Recurrence**								
no	780 (87.25)	179 (86.06)	Ref.		471 (89.54)	650 (84.86)	Ref.	
yes	114 (12.75)	29 (13.94)	1.19 (−4.00, 6.38)	0.65	55 (10.46)	116 (15.14)	4.69 (1.04, 8.33)	0.02

**Table 4:** Odds ratios of the associations between low visual ITH and molecular indicators of heterogeneity in CBCS and TCGA (original only). Clonal/multi-clonal classes are defined based on the number of observed tumor subclonal populations, and TP53 mutations were identified using targeted (CBCS) or whole-exome sequencing (TCGA).

	Clonal		Multi-Clonal		Overall OR (95% CI)
	**Low Visual ITH**	**High Visual ITH**	**Low Visual ITH**	**High Visual ITH**	
Original	113 (66%)	77 (55%)	59 (34%)	62 (45%)	1.54 (0.98, 2.45)
Stromal	110 (66%)	80 (55%)	56 (34%)	65 (45%)	1.60 (1.01, 2.53)
Epithelial	107 (62%)	83 (60%)	66 (38%)	55 (40%)	1.07 (0.68, 1.70)
TCGA	96 (53%)	426 (58%)	86 (47%)	307 (42%)	0.80 (0.58, 1.12)
	**TP53 Mutation**		**No TP53 Mutation**		**Overall OR (95% CI)**
	**Low Visual ITH**	**High Visual ITH**	**Low Visual ITH**	**High Visual ITH**	
Original	78 (43%)	44 (27%)	105 (57%)	117 (73%)	1.98 (1.26, 3.13)
Stromal	74 (41%)	48 (29%)	105 (59%)	117 (71%)	1.72 (1.10, 2.70)
Epithelial	71 (39%)	51 (32%)	113 (61%)	109 (68%)	1.34 (0.87, 2.10)
TCGA	64 (35%)	222 (30%)	118 (65%)	511 (70%)	1.25 (0.89, 1.75)

## Data Availability

The Carolina Breast Cancer Study is actively following patients under an IRB-approved protocol that does not permit data sharing on public websites. However, we share data through an IRB-approved data use agreement system, as described on our website (https://unclineberger.org/cbcs/for-researchers/), and requests for biospecimen and sequencing data can be made by contacting the study authors. The results published here are in whole or in part based upon data from the Cancer Genome Atlas managed by the NCI and NHGRI (dbGaP accession phs000178).

## References

[1] Gerlinger M., Rowan A.J., Horswell S., Larkin J., Endesfelder D., Gronroos E., Martinez P., Matthews N., Stewart A., Tarpey P. (2012). Intratumor heterogeneity and branched evolution revealed by multiregion sequencing. N. Engl. J. Med..

[2] Swanton C. (2012). Intratumor heterogeneity: Evolution through space and time. Cancer Res..

[3] Allott E.H., Geradts J., Sun X., Cohen S.M., Zirpoli G.R., Khoury T., Bshara W., Chen M., Sherman M.E., Palmer J.R. (2016). Intratumoral heterogeneity as a source of discordance in breast cancer biomarker classification. Breast Cancer Res..

[4] Lindström L.S., Yau C., Czene K., Thompson C.K., Hoadley K.A., Van’t Veer L.J., Balassanian R., Bishop J.W., Carpenter P.M., Chen Y.Y. (2018). Intratumor heterogeneity of the estrogen receptor and the long-term risk of fatal breast cancer. J. Natl. Cancer Inst..

[5] McGranahan N., Swanton C. (2017). Clonal Heterogeneity and Tumor Evolution: Past, Present, and the Future. Cell.

[6] Gatenby R.A., Silva A.S., Gillies R.J., Frieden B.R. (2009). Adaptive therapy. Cancer Res..

[7] Dentro S.C., Leshchiner I., Haase K., Tarabichi M., Wintersinger J., Deshwar A.G., Yu K., Rubanova Y., Macintyre G., Demeulemeester J. (2021). Characterizing genetic intra-tumor heterogeneity across 2,658 human cancer genomes. Cell.

[8] Van Keymeulen A., Lee M.Y., Ousset M., Brohée S., Rorive S., Giraddi R.R., Wuidart A., Bouvencourt G., Dubois C., Salmon I. (2015). Reactivation of multipotency by oncogenic PIK3CA induces breast tumour heterogeneity. Nature.

[9] Li Z., Seehawer M., Polyak C. (2022). Untangling the web of intratumour heterogeneity. Nat. Cell Biol..

[10] Marusyk A., Janiszewska M., Polyak C. (2020). Intratumor heterogeneity: The Rosetta stone of therapy resistance. Cancer Cell.

[11] Fassler D.J., Torre-Healy L.A., Gupta R., Hamilton A.M., Kobayashi S., Van Alsten S.C., Zhang Y., Kurc T., Moffitt R.A., Troester M.A. (2022). Spatial characterization of tumor-infiltrating lymphocytes and breast cancer progression. Cancers.

[12] Romero-Cordoba S., Meneghini E., Sant M., Iorio M.V., Sfondrini L., Paolini B., Agresti R., Tagliabue E., Bianchi F. (2019). Decoding immune heterogeneity of triple negative breast cancer and its association with systemic inflammation. Cancers.

[13] Liegmann A.S., Heselmeyer-Haddad K., Lischka A., Hirsch D., Chen W.D., Torres I., Gemoll T., Rody A., Thorns C., Gertz E.M. (2021). Single cell genetic profiling of tumors of breast cancer patients aged 50 years and older reveals enormous intratumor heterogeneity independent of individual prognosis. Cancers.

[14] Jackson H.W., Fischer J.R., Zanotelli V.R., Ali H.R., Mechera R., Soysal S.D., Moch H., Muenst S., Varga Z., Weber W.P. (2020). The single-cell pathology landscape of breast cancer. Nature.

[15] Carey L.A., Perou C.M., Livasy C.A., Dressler L.G., Cowan D., Conway K., Karaca G., Troester M.A., Tse C.K., Edmiston S. (2006). Race, breast cancer subtypes, and survival in the Carolina Breast Cancer Study. JAMA.

[16] O’Brien K.M., Cole S.R., Tse C.K., Perou C.M., Carey L.A., Foulkes W.D., Dressler L.G., Geradts J., Millikan R.C. (2010). Intrinsic breast tumor subtypes, race, and long-term survival in the Carolina Breast Cancer Study. Clin. Cancer Res..

[17] Newman B., Moorman P.G., Millikan R., Qaqish B.F., Geradts J., Aldrich T.E., Liu E.T. (1995). The Carolina Breast Cancer Study: Integrating population-based epidemiology and molecular biology. Breast Cancer Res. Treat..

[18] Razzaghi H., Troester M.A., Gierach G.L., Olshan A.F., Yankaskas B.C., Millikan R.C. (2012). Mammographic density and breast cancer risk in White and African American Women. Breast Cancer Res. Treat..

[19] Conway K., Parrish E., Edmiston S.N., Tolbert D., Tse C.K., Moorman P., Newman B., Millikan R.C. (2007). Risk factors for breast cancer characterized by the estrogen receptor alpha A908G (K303R) mutation. Breast Cancer Res..

[20] Millikan R.C., Newman B., Tse C.K., Moorman P.G., Conway K., Smith L.V., Labbok M.H., Geradts J., Bensen J.T., Jackson S. (2008). Epidemiology of basal-like breast cancer. Breast Cancer Res. Treat..

[21] Network C.G.A. (2012). Comprehensive molecular portraits of human breast tumours. Nature.

[22] Ciriello G., Gatza M.L., Beck A.H., Wilkerson M.D., Rhie S.K., Pastore A., Zhang H., McLellan M., Yau C., Kandoth C. (2015). Comprehensive molecular portraits of invasive lobular breast cancer. Cell.

[23] Macenko M., Niethammer M., Marron J.S., Borland D., Woosley J.T., Guan X., Schmitt C., Thomas N.E. A Method for Normalizing Histology Slides for Quantitative Analysis. Proceedings of the 2009 IEEE International Symposium on Biomedical Imaging: From Nano to Macro.

[24] Klimov S., Miligy I.M., Gertych A., Jiang Y., Toss M.S., Rida P., Ellis I.O., Green A., Krishnamurti U., Rakha E.A. (2019). A whole slide image-based machine learning approach to predict ductal carcinoma in situ (DCIS) recurrence risk. Breast Cancer Res..

[25] Bankhead P., Loughrey M.B., Fernández J.A., Dombrowski Y., McArt D.G., Dunne P.D., McQuaid S., Gray R.T., Murray L.J., Coleman H.G. (2017). QuPath: Open source software for digital pathology image analysis. Sci. Rep..

[26] Simonyan K., Zisserman A. (2014). Very deep convolutional networks for large-scale image recognition. arXiv.

[27] Beck A.H., Sangoi A.R., Leung S., Marinelli R.J., Nielsen T.O., Van De Vijver M.J., West R.B., Van De Rijn M., Koller D. (2011). Systematic analysis of breast cancer morphology uncovers stromal features associated with survival. Sci. Transl. Med..

[28] Chang H., Fontenay G.V., Han J., Cong G., Baehner F.L., Gray J.W., Spellman P.T., Parvin B. (2011). Morphometic analysis of TCGA glioblastoma multiforme. BMC Bioinform..

[29] Miedema J., Marron J.S., Niethammer M., Borland D., Woosley J., Coposky J., Wei S., Reisner H., Thomas N.E. (2012). Image and statistical analysis of melanocytic histology. Histopathology.

[30] Cooper L.A., Kong J., Gutman D.A., Wang F., Gao J., Appin C., Cholleti S., Pan T., Sharma A., Scarpace L. (2012). Integrated morphologic analysis for the identification and characterization of disease subtypes. J. Am. Med. Inform. Assoc..

[31] Hou L., Samaras D., Kurc T.M., Gao Y., Davis J.E., Saltz J.H. Patch-Based Convolutional Neural Network for Whole Slide Tissue Image Classification. Proceedings of the IEEE Conference on Computer Vision and Pattern Recognition.

[32] Xu J., Luo X., Wang G., Gilmore H., Madabhushi A. (2016). A deep convolutional neural network for segmenting and classifying epithelial and stromal regions in histopathological images. Neurocomputing.

[33] Deng J., Dong W., Socher R., Li L.J., Li K., Fei-Fei L. Imagenet: A Large-Scale Hierarchical Image Database. Proceedings of the 2009 IEEE Conference on Computer Vision and Pattern Recognition.

[34] Jain A.K., Dubes R.C. (1988). Algorithms for Clustering Data.

[35] Ward J.H. (1963). Hierarchical grouping to optimize an objective function. J. Am. Stat. Assoc..

[36] Mose L.E., Perou C.M., Parker J.S. (2019). Improved indel detection in DNA and RNA via realignment with ABRA2. Bioinformatics.

[37] Benjamin D., Sato T., Cibulskis K., Getz G., Stewart C., Lichtenstein L. (2019). Calling Somatic SNVs and Indels with Mutect2. bioRxiv.

[38] Kim S., Scheffler K., Halpern A.L., Bekritsky M.A., Noh E., Källberg M., Chen X., Kim Y., Beyter D., Krusche P. (2018). Strelka2: Fast and accurate calling of germline and somatic variants. Nat. Methods.

[39] Talevich E., Shain A.H., Botton T., Bastian B.C. (2016). CNVkit: Genome-wide copy number detection and visualization from targeted DNA sequencing. PLoS Comput. Biol..

[40] Gillis S., Roth A. (2020). PyClone-VI: Scalable inference of clonal population structures using whole genome data. BMC Bioinform..

[41] Parker J.S., Mullins M., Cheang M.C., Leung S., Voduc D., Vickery T., Davies S., Fauron C., He X., Hu Z. (2009). Supervised risk predictor of breast cancer based on intrinsic subtypes. J. Clin. Oncol..

[42] Hamilton A.M., Hurson A.N., Olsson L.T., Walens A., Nsonwu-Farley J., Kirk E.L., Abdou Y., Downs-Canner S., Serody J.S., Perou C.M. (2022). The landscape of immune microenvironments in racially diverse breast cancer patients. Cancer Epidemiol. Biomarkers Prev..

[43] Olsson L.T., Williams L.A., Midkiff B.R., Kirk E.L., Troester M.A., Calhoun B.C. (2022). Quantitative analysis of breast cancer tissue composition and associations with tumor subtype. Hum. Pathol..

[44] Razavi P., Chang M.T., Xu G., Bandlamudi C., Ross D.S., Vasan N., Cai Y., Bielski C.M., Donoghue M.T., Jonsson P. (2018). The genomic landscape of endocrine-resistant advanced breast cancers. Cancer Cell.

[45] Angus L., Smid M., Wilting S.M., van Riet J., Van Hoeck A., Nguyen L., Nik-Zainal S., Steenbruggen T.G., Tjan-Heijnen V.C., Labots M. (2019). The genomic landscape of metastatic breast cancer highlights changes in mutation and signature frequencies. Nat. Genet..

[46] Keenan T., Moy B., Mroz E.A., Ross K., Niemierko A., Rocco J.W., Isakoff S., Ellisen L.W., Bardia A. (2015). Comparison of the genomic landscape between primary breast cancer in African American versus white women and the association of racial differences with tumor recurrence. J. Clin. Oncol..

[47] Pereira B., Chin S.F., Rueda O.M., Vollan H.K.M., Provenzano E., Bardwell H.A., Pugh M., Jones L., Russell R., Sammut S.J. (2016). The somatic mutation profiles of 2,433 breast cancers refine their genomic and transcriptomic landscapes. Nat. Commun..

[48] Almendro V., Marusyk A., Polyak K. (2013). Cellular heterogeneity and molecular evolution in cancer. Annu. Rev. Pathol. Mech. Dis..

[49] Graf J.F., Zavodszky M.I. (2017). Characterizing the heterogeneity of tumor tissues from spatially resolved molecular measures. PLoS ONE.

[50] Lineweaver C.H., Bussey K.J., Blackburn A.C., Davies P.C. (2021). Cancer progression as a sequence of atavistic reversions. BioEssays.

[51] Budczies J., Bockmayr M., Denkert C., Klauschen F., Lennerz J.K., Gyorffy B., Dietsel M., Loibl S., Weichert W., Stenzinger A. (2015). Classical pathology and mutational load of breast cancer— Integration of two worlds. J. Pathol. Clin. Res..

[52] Donehower L.A., Soussi T., Korkut A., Liu Y., Schultz A., Cardenas M., Li X., Babur O., Hsu T.K., Lichtarge O. (2019). Integrated analysis of TP53 gene and pathway alterations in the cancer genome atlas. Cell Rep..

[53] Fuller A.M., Yang L., Hamilton A.M., Pirone J.R., Oldenburg A.L., Troester M.A. (2021). Epithelial p53 status modifies stromal-epithelial interactions during basal-like breast carcinogenesis. J. Mammary Gland. Biol. Neoplasia.

[54] Hamilton A.M., Van Alsten S.C., Gao X., Nsonwu-Farley J., Calhoun B.C., Love M.I., Troester M.A., Hoadley K.A. (2023). Incorporating RNA-based risk scores for genomic instability to predict breast cancer recurrence and immunogenicity in a diverse population. Cancer Res. Commun..

[55] Tarabichi M., Salcedo A., Deshwar A.G., Leathlobhair M.N., Wintersinger J., Wedge D.C., Van Loo P., Morris Q.D., Boutros P.C. (2021). A practical guide to cancer subclonal reconstruction from DNA sequencing. Nat. Methods.

